# Socioeconomic Status, Trauma, Cognitive Function, Impulsivity, Reward Salience, and Future Substance Use: Role of Left Caudate Connectivity with the Cingulo-Opercular Network

**Published:** 2025-02-24

**Authors:** Shervin Assari, Hossein Zare, Golnoush Akhlaghipour, Mario F Mendez

**Affiliations:** 1 Department of Internal Medicine, Charles R. Drew University of Medicine and Science, Los Angeles, CA, USA; 2 Department of Urban Public Health, Charles R. Drew University of Medicine and Science, Los Angeles, CA, USA; 3 Marginalization-Related Diminished Returns (MDRs) Center, Los Angeles, CA, USA; 4 Department of Health Policy and Management, Johns Hopkins Bloomberg School of Public Health, Baltimore, MD, USA; 5 School of Business, University of Maryland Global Campus (UMGC), Adelphi, USA; 6 Department of Neurology, University of California Los Angeles (UCLA), Los Angeles, CA, USA; 7 Department of Psychiatry & Biobehavioral Sciences, University of California Los Angeles (UCLA), Los Angeles, CA, USA

**Keywords:** Resting-State Functional Connectivity, Left Caudate, Cingulo-Opercular Network, Socioeconomic Status, Trauma, Cognitive Function, Impulsivity, Depression, Substance Use

## Abstract

**Background:**

While understanding how corticostriatal connectivity is associated with socioeconomic status (SES), trauma exposure, cognitive function, reward salience, impulsivity, and future substance use is essential to identifying neurobiological pathways that contribute to health disparities and behavioral outcomes, very few studies have tested the role of left caudate resting-state functional connectivity (rsFC) with the cingulo-opercular network as a proxy of corticostriatal connectivity in social, cognitive, and behavioral processes.

**Objective:**

This study investigates the associations between left caudate-cingulo-opercular connectivity and multiple biopsychosocial domains, including low SES, high trauma exposure (financial and life events), cognitive function, reward salience, impulsivity, depression, and future substance use (tobacco and marijuana use).

**Methods:**

Resting-state functional magnetic resonance imaging (rs-fMRI) data were analyzed to assess connectivity between the left caudate and the cingulo-opercular network. Data on socioeconomic status, trauma exposure, cognitive performance, and mental health were collected from participants. Future substance use behaviors were evaluated through longitudinal follow-ups. Correlation and regression analyses were conducted to examine relationships between corticostriatal connectivity and the targeted domains.

**Results:**

Corticostriatal hypoconnectivity was associated with lower SES, higher trauma exposure, poorer cognitive function, heightened reward salience, higher impulsivity, and history of depression. Additionally, corticostriatal hypoconnectivity at baseline predicted future tobacco and marijuana use during follow-up years.

**Conclusion:**

Corticostriatal hypoconnectivity, particularly the rsFC between the left caudate and the cingulo-opercular network, may represent a potential mechanism linking a wide range of social, emotional, and behavioral problems in youth. These findings suggest that corticostriatal hypoconnectivity could serve as a neurobiological marker for identifying individuals at risk for depression, low cognitive function, high reward salience, impulsivity, and substance use, emphasizing the interplay between socioeconomic and neurocognitive factors in shaping behavioral health trajectories.

## 1. Introduction

Resting-state functional connectivity (rsFC) refers to the temporal correlation of spontaneous neural activity between distinct brain regions or networks when an individual is not engaged in any specific task [1]. RsFC provides valuable insights into intrinsic brain organization and is widely used to examine how neural networks interact in both healthy and clinical populations [2,3]. Studies utilizing rsFC have identified patterns of connectivity that underlie cognitive and emotional processes, highlighting its potential in understanding mental health outcomes [4]. Disruptions in rsFC have been linked to various neuropsychiatric disorders, including depression, anxiety, and substance use, making it a critical area of investigation in developmental neuroscience [5].

Corticostriatal connectivity, which encompasses functional interactions between the cortex and the striatum, is particularly important in regulating cognitive control, emotion, and reward-related behaviors [6–9]. This connectivity plays a vital role in goal-directed behavior, habit formation, and emotional regulation [10–12]. The corticostriatal pathway has been implicated in neurodevelopmental disorders, addiction, and psychiatric conditions, with variations in connectivity patterns observed across different socioeconomic and environmental contexts [13–15]. Understanding the factors that influence corticostriatal connectivity is crucial for identifying neurobiological mechanisms that contribute to health disparities and behavioral outcomes.

The caudate nucleus, a major component of the striatum, is involved in various cognitive functions, including decision-making, learning, and reward processing [16–19]. It plays a pivotal role in modulating goal-directed behavior and is integral to the corticostriatal circuitry [16,20–24]. Structural and functional abnormalities in the caudate have been associated with impulsivity, cognitive deficits, and increased vulnerability to substance use disorders. The caudate’s connectivity with cortical regions, particularly those involved in executive function and emotional regulation, provides critical insights into individual differences in cognitive and affective processing.

In 1989, Mendez et al. [25] was among the first to highlight the role of the caudate nucleus in behavior. The study examined 12 patients with caudate lesions—11 unilateral and one bilateral—and documented significant behavioral and cognitive changes. Patients exhibited acute behavioral alterations, including apathy, disinhibition, or major affective disturbances. The nature of personality changes was found to be associated with the size and location of the lesion within the caudate, rather than its laterality. A subset of seven patients was further assessed using neuropsychological tests and compared with matched controls. Those with caudate lesions demonstrated deficits in tasks requiring planning and sequencing, exhibited short attention spans, and showed reduced free recall for episodic and semantic information, despite intact recognition memory. Similar cognitive and behavioral impairments have been observed in individuals with early Huntington’s disease, frontal lobe damage, and caudate lesions in animal models, suggesting disruptions in specific frontal-caudate circuits. These findings provided early evidence of the caudate’s role in prefrontal-mediated behaviors and the conceptual integration of memory. Subsequent studies have since reinforced these findings, further establishing the role of caudate dysfunction in behavioral, cognitive, and memory disturbances [25–28].

A meta-analytic connectivity modeling (MACM) approach [29] was utilized to examine the functional connectivity of the human caudate. The study aimed to: (1) construct a model of functional connectivity, (2) assess whether meta-analytic techniques are sensitive enough to identify behavioral domain specificity within region-specific connectivity networks, and (3) compare meta-analytically derived segmentation with structural connectivity parcellation obtained through diffusion tensor imaging (DTI). The findings indicated that behavioral filtering distinguished cognition- and emotion-related structures and networks, primarily concentrated in the head of the caudate nucleus, while regions associated with perception and action were localized to the body of the caudate. These results align with earlier histological studies in nonhuman primates and postmortem research in humans. Additionally, DTI findings supported the utility of MACM in detecting both direct and indirect connectivity patterns. Overall, the study provided further validation for MACM while demonstrating its potential to extract behavioral domain-specific functional connectivity [29].

The human dorsal striatum serves as the primary input region to the basal ganglia [21,30–33]. Comprising the caudate and putamen, it receives projections from nearly all cortical areas, except for the primary visual, auditory, and olfactory cortices [34]. Structural, functional, and connectivity alterations in the caudate nucleus have been observed in various neuropsychiatric conditions, including depression [35], attention deficit disorders [36], and substance addiction [37,38].

In their review paper, Jessica Grahn and colleagues explored the distinct functional roles of the caudate nucleus and the putamen. Their analysis integrated findings from multiple domains, including anatomical studies of corticostriatal pathways, neuroimaging research on healthy individuals, patient studies examining cognitive deficits, and animal research on behavioral regulation. They concluded that the caudate nucleus plays a key role in facilitating behavior by activating appropriate action schemas and selecting sub-goals based on action-outcome evaluations—both essential for goal-directed behavior. In contrast, putamen was primarily associated with cognitive processes related to stimulus-response learning and habit formation. Their findings support a modular framework of striatal function, aligning with hierarchical models of cortico-striatal processing, in which the ventral striatum is linked to motivation, the caudate to cognitive planning, and the putamen to sensorimotor execution, collectively enabling adaptive goal-directed behavior [21].

The cingulo-opercular network [39–41]is a core cognitive control network that includes regions such as the anterior cingulate cortex (ACC), insula, and frontal operculum [42–44]. This network is responsible for maintaining task performance, error monitoring, and adaptive control in response to changing environmental demands [45–47]. It has been shown to be involved in sustained attention, emotional regulation, and self-monitoring processes. Alterations in cingulo-opercular network connectivity have been linked to mood disorders, impulsivity, and substance use behaviors, highlighting its relevance in understanding the interplay between brain function and psychosocial factors.

RsFC between the left caudate and the cingulo-opercular network, here referred to as corticostriatal connectivity, may play a role in cognitive and affective processes. This connectivity is linked to various psychosocial and behavioral factors, including socioeconomic status (SES), trauma exposure, cognitive function, reward salience, impulsivity, and future substance use. Investigating these associations can provide insights into how environmental and individual factors interact with neurobiological mechanisms to influence mental health and behavioral outcomes over time.

This study investigates the associations between left caudate-cingulo-opercular connectivity and multiple domains, including low SES, high trauma exposure (financial and life events), cognitive function, reward salience, impulsivity, depression, and future substance use (tobacco and marijuana use). The following hypotheses were examined: (1) lower SES and higher trauma exposure will be associated with altered corticostriatal connectivity, (2) variations in corticostriatal connectivity will predict differences in cognitive function, impulsivity, and reward salience, and (3) disrupted corticostriatal connectivity at baseline will be linked to increased risk of future tobacco and marijuana use. These findings aim to contribute to a better understanding of the neurobiological underpinnings of health disparities and substance use vulnerability in youth.

## 2. Methods

### 2.1. Study Design and Participants

This study utilized data from the Adolescent Brain Cognitive Development (ABCD) Study, a large, ongoing longitudinal cohort designed to investigate the developmental trajectories of brain function and behavior in youth across the United States. The sample included participants with complete resting-state functional magnetic resonance imaging (rs-fMRI) data, alongside comprehensive measures of socioeconomic status (SES), trauma exposure, cognitive function, reward salience, impulsivity, and substance use behaviors. Inclusion criteria required participants to have no history of neurological disorders and to have complete data on key study variables. Participants provided written informed consent/assent, and ethical approval was obtained from institutional review boards at participating sites.

### 2.2. Measures

#### 2.2.1. Neuroimaging Data

Resting-state functional connectivity (rsFC) was assessed using rs-fMRI data from the ABCD study. Preprocessed neuroimaging data were used to examine connectivity between the left caudate and the cingulo-opercular network, which includes key regions such as the anterior cingulate cortex, insula, and frontal operculum. Standard preprocessing steps, including motion correction, spatial normalization, and nuisance regression, were applied to ensure data quality. Connectivity values were derived using a seed-based approach, with the left caudate as the seed region and averaged time-series data extracted from the cingulo-opercular network.

#### 2.2.2. Socioeconomic Status (SES)

SES was measured using several indicators, including parental education, household income, income-to-needs ratio, and financial strain. Higher values on these variables indicated greater socioeconomic resources, while lower values reflected economic hardship.

#### 2.2.3. Trauma Exposure (Stress)

Trauma exposure was assessed using self-reported measures capturing financial strain and life events. Participants reported experiences related to financial hardships, housing instability, and other major life stressors.

#### 2.2.4. Cognitive Function

Cognitive performance was evaluated using the NIH Toolbox, which includes measures of executive function, processing speed, and working memory. Composite scores for crystallized and fluid intelligence were used to represent overall cognitive ability.

#### 2.2.5. Impulsivity and Reward Salience

Impulsivity was assessed using validated behavioral measures of negative and positive urgency, while reward salience was measured through self-reports of drive and sensation-seeking tendencies.

#### 2.2.6. Mental Health and Substance Use

Depression history was assessed using diagnostic interviews and self-reports of prior major depressive disorder (MDD) diagnoses. Future substance use, including tobacco and marijuana use, was evaluated through longitudinal follow-up assessments at subsequent data waves.

### 2.3. Statistical Analysis

#### 2.3.1. Structural Equation Modeling (SEM)

To examine the complex relationships between SES, trauma exposure, rsFC, and behavioral outcomes, structural equation modeling (SEM) was employed. The model included the following components:

- **Predictors:**
  - Socioeconomic status (SES)
  - Trauma exposure (Stress)
- **Mediator:**
  - Left caudate-cingulo-opercular resting-state functional connectivity (rsFC)
- **Latent Outcome Factors:**
  - Cognition (measured via various cognitive function tests)
  - Impulsivity (including measures of positive and negative urgency)
  - Substance use (future tobacco and marijuana use)
- **Observed Outcome Variables:**
  - Body Mass Index (BMI)
  - Grade Point Average (GPA)
  - Depression history (MDD)

### 2.4. Statistical Analysis

The SEM model was estimated using maximum likelihood estimation with bootstrapping to assess indirect effects. Model fit was evaluated using common fit indices, including the Comparative Fit Index (CFI), Tucker-Lewis Index (TLI), and the Root Mean Square Error of Approximation (RMSEA). All analyses were conducted using Mplus software, and significance was set at *p* < 0.05. Descriptive statistics were used to summarize the characteristics of the study sample. Bivariate correlations were conducted to examine the associations between left caudate-cingulo-opercular connectivity and the biopsychosocial variables of interest. Multiple linear regression models were performed to adjust for potential confounders, including age, sex, and scanner site, to assess the independent effects of rsFC on future behavioral outcomes.

### 2.5. Ethical Considerations

Data used in this study were obtained from the publicly available ABCD Study dataset, and all analyses adhered to data use agreements and ethical guidelines. The study was conducted in accordance with the Declaration of Helsinki, and participant confidentiality was maintained through data de-identification procedures.

## 3. Results

As shown in Table 1, bivariate correlation analyses were conducted to examine the associations between left caudate-cingulo-opercular resting-state functional connectivity (rsFC) and a range of biopsychosocial factors, including socioeconomic status (SES), trauma exposure, cognitive function, impulsivity, and future substance use. All reported p-values were statistically significant.

### 3.1. Associations Between rsFC and Socioeconomic Indicators

The results showed small but statistically significant correlations between rsFC and several socioeconomic factors. Parental education (r = 0.02), income-to-needs ratio (r = 0.02), and household income normalized to $50,000 (r = 0.04) were positively associated with rsFC, suggesting that higher SES may be linked to greater corticostriatal connectivity. Conversely, financial strain (r = −0.03) was negatively associated with rsFC, indicating a possible adverse effect of economic hardship on neural connectivity.

### 3.2. Associations Between rsFC and Trauma and Discrimination

A negative correlation was observed between rsFC and both trauma exposure (r = −0.03) and perceived discrimination (r = −0.03). These findings suggest that greater exposure to life stressors and discrimination may be linked to lower corticostriatal connectivity, highlighting the potential neurobiological impact of social adversity.

### 3.3. Associations Between rsFC and Cognitive Function

Significant positive associations were found between rsFC and various measures of cognitive performance. Cognitive domains such as GPA (r = 0.03), card sorting (r = 0.03), fluid cognition (r = 0.03), pattern recognition (r = 0.03), and overall cognitive composition (r = 0.03) showed positive relationships with rsFC. These results suggest that greater connectivity may be associated with better cognitive performance across multiple domains.

### 3.4. Associations Between rsFC and Behavioral and Emotional Factors

Negative urgency (r = −0.02) and positive urgency (r = −0.03), reflecting impulsivity traits, were negatively correlated with rsFC, suggesting that lower connectivity may be associated with higher impulsivity. Past major depressive disorder (MDD) diagnosis was also negatively associated with rsFC (r = −0.02), indicating a potential link between lower connectivity and a history of depression. Drive, which reflects goal-directed motivation, showed a weak negative association with rsFC (r = −0.01).

### 3.5. Associations Between rsFC and Future Substance Use

Baseline rsFC was negatively associated with future tobacco use (r = −0.03), marijuana use (r = −0.01), and combined tobacco and marijuana use (r = −0.01). When analyzed separately, future tobacco-only use (r = −0.02) and future marijuana-only use (r = −0.01) also exhibited weak negative correlations with rsFC. These findings suggest that lower corticostriatal connectivity may be associated with an increased risk of future substance use behaviors.

### 3.6. Summary of Results

The structural equation modeling (SEM) results provided insights into the relationships between resting-state functional connectivity (rsFC) of the left caudate-cingulo-opercular network, socioeconomic status (SES), stress, and various behavioral and health outcomes, including BMI, past major depressive disorder (MDD), GPA, substance use, cognitive function, and impulsivity.

#### 3.6.1. Resting-State Functional Connectivity (rsFC)

Stress was significantly and positively associated with rsFC (β = 0.229, p < 0.001), indicating that higher stress levels were linked to greater corticostriatal connectivity.

SES was negatively associated with rsFC (β = −0.983, p < 0.001), suggesting that lower SES is related to decreased connectivity within this network.

#### 3.6.2. Body Mass Index (BMI)

rsFC showed a small but significant negative association with BMI (β = −0.034, p = 0.029), indicating that lower corticostriatal connectivity may be linked to higher BMI.

Stress was negatively associated with BMI (β = −0.943, p < 0.001), while SES was positively associated with BMI (β = 0.901, p < 0.001), suggesting that higher SES is linked to increased BMI, whereas stress contributes to lower BMI.

#### 3.6.3. Past Major Depressive Disorder (MDD)

No significant associations were observed between rsFC and past MDD (β = −0.025, p = 0.146), stress (β = −0.155, p = 0.647), or SES (β = 0.090, p = 0.824), indicating that these factors may not have a strong direct effect on past depressive episodes.

#### 3.6.4. Grade Point Average (GPA)

rsFC was marginally associated with GPA (β = 0.040, p = 0.098), suggesting a potential but weak relationship between greater connectivity and academic performance.

Neither stress (β = −0.101, p = 0.746) nor SES (β = −0.184, p = 0.648) showed significant associations with GPA.

#### 3.6.5. Future Substance Use

rsFC was not significantly associated with future substance use (β = −0.023, p = 0.174), nor were stress (β = 0.044, p = 0.905) and SES (β = 0.252, p = 0.500), suggesting that other factors may play a more critical role in predicting substance use behaviors.

#### 3.6.6. Cognitive Function

rsFC was positively associated with cognitive function (β = 0.013, p < 0.001), indicating that higher corticostriatal connectivity may contribute to better cognitive performance.

Both stress (β = 0.415, p < 0.001) and SES (β = 0.583, p < 0.001) were significantly positively associated with cognitive function, suggesting that higher SES and stress levels may enhance cognitive abilities in this population.

#### 3.6.7. Impulsivity

A small but significant positive association was found between rsFC and impulsivity (β = 0.013, p = 0.034), indicating that greater connectivity may be linked to higher impulsivity.

Stress (β = 0.671, p < 0.001) and SES (β = 0.297, p < 0.001) were also positively associated with impulsivity, suggesting that both social and biological factors contribute to impulsivity levels.

Table 3 shows the results of the measurement section of the SEM. Standardized factor loadings for all observed indicators were statistically significant (*p* < 0.001), demonstrating strong relationships between the latent constructs and their respective indicators.

#### 3.6.8. Substance Use

The latent factor for substance use was measured using indicators of tobacco and marijuana use. Both indicators demonstrated significant positive loadings, with tobacco use showing a higher loading (B = 0.767, SE = 0.0191, 95% CI [0.730, 0.805], *p* < 0.001) compared to marijuana use (B = 0.614, SE = 0.0161, 95% CI [0.583, 0.646], *p* < 0.001). These findings suggest that tobacco use contributes more strongly to the overall substance use construct.

#### 3.6.9. Cognitive Function

Cognition was represented by several indicators, all of which demonstrated strong loadings onto the latent factor. Total cognitive composition (B = 0.999, SE = 0.0000, 95% CI [0.998, 0.999], *p* < 0.001) had the highest loading, indicating nearly perfect alignment with the latent construct. Other cognitive indicators, such as crystalized cognition (B = 0.995, SE = 0.0001, 95% CI [0.995, 0.996], *p* < 0.001) and fluid composition (B = 0.992, SE = 0.0002, 95% CI [0.992, 0.992], *p* < 0.001), also showed high loadings. The relatively lower loading of pattern recognition (B = 0.975, SE = 0.0005, 95% CI [0.974, 0.976], *p* < 0.001) suggests a slightly weaker but still significant contribution to the overall cognitive function construct.

#### 3.6.10. Impulsivity

Impulsivity was assessed using measures of negative and positive urgency. Negative urgency showed a stronger association with the latent impulsivity factor (B = 0.980, SE = 0.0008, 95% CI [0.978, 0.981], *p* < 0.001) compared to positive urgency (B = 0.957, SE = 0.0013, 95% CI [0.954, 0.960], *p* < 0.001), indicating that negative urgency may play a more central role in impulsivity-related behaviors.

#### 3.6.11. Stress

The latent stress construct was measured by trauma, financial difficulty, and discrimination. Discrimination exhibited the highest factor loading (B = 0.918, SE = 0.0020, 95% CI [0.914, 0.922], *p* < 0.001), suggesting that perceived discrimination is a key contributor to the overall stress experienced by participants. Trauma (B = 0.406, SE = 0.0074, 95% CI [0.391, 0.421], *p* < 0.001) and financial difficulty (B = 0.347, SE = 0.0077, 95% CI [0.332, 0.362], *p* < 0.001) had lower loadings, indicating that while significant, they may contribute less to the overall stress experience.

#### 3.6.12. Socioeconomic Status (SES)

SES was represented by indicators of parental education, income-to-needs ratio, marital status, and income level. Education showed the strongest loading (B = 0.999, SE = 0.0002, 95% CI [0.999, 1.000], *p* < 0.001), indicating a nearly perfect representation of SES through educational attainment. Income-to-needs ratio (B = 0.907, SE = 0.0017, 95% CI [0.904, 0.910], *p* < 0.001) and marital status (B = 0.849, SE = 0.0027, 95% CI [0.844, 0.854], *p* < 0.001) also significantly contributed to the SES construct, while normalized income (B = 0.832, SE = 0.0029, 95% CI [0.827, 0.838], *p* < 0.001) had the lowest loading among the SES indicators.

## 4. Discussion

This study aimed to explore the potential associations between left caudate-cingulo-opercular resting-state functional connectivity (rsFC), referred to as corticostriatal connectivity, and various biopsychosocial domains, including socioeconomic status (SES), trauma exposure, cognitive function, reward salience, impulsivity, depression, and future substance use. It was hypothesized that corticostriatal connectivity may be associated with SES and stress-related factors, which could, in turn, influence cognitive, emotional, and behavioral outcomes such as impulsivity and future substance use.

Overall, the findings suggest that socioeconomic status and stress play critical roles in shaping brain connectivity and cognitive outcomes, with significant associations observed between rsFC and cognitive function, impulsivity, and BMI. While rsFC was not significantly associated with past depression or future substance use, its relationships with cognitive and behavioral measures underscore its potential relevance in understanding health disparities in youth.

The findings suggest that socioeconomic status and stress may play important roles in shaping corticostriatal connectivity and its downstream effects on cognition and behavior. Lower connectivity between the left caudate and the cingulo-opercular network was found to be associated with socioeconomic and environmental factors and poorer cognitive function. However, rsFC did not demonstrate significant associations with past depression or future substance use, suggesting that other contributing factors may play a more significant role in these outcomes.

The results suggest that lower SES may be associated with reduced corticostriatal connectivity, which could have implications for cognitive and emotional development. Prior research has indicated that low SES is linked to structural and functional changes in brain regions involved in executive functioning and decision-making. Potential mechanisms underlying this relationship may include chronic stress exposure, limited access to cognitive resources, and environmental adversities. Similarly, stress appeared to be associated with increased corticostriatal connectivity, suggesting potential compensatory neural responses to prolonged stress exposure.

The study findings suggest that higher corticostriatal connectivity may be associated with better cognitive function, supporting the potential role of this neural network in executive functioning and cognitive flexibility. These results align with previous research that has highlighted the caudate’s involvement in goal-directed behavior and cognitive processing. The findings highlight the importance of addressing socioeconomic and environmental disparities that may negatively impact neural connectivity and cognitive resilience in at-risk youth.

Findings indicate that greater corticostriatal connectivity may be linked to higher impulsivity, suggesting that increased connectivity could reflect compensatory mechanisms or neural inefficiencies related to self-regulation. Although previous studies have identified corticostriatal pathways as critical for impulse control and reward processing, further research is needed to clarify the directionality and potential moderators of these relationships.

While bivariate correlations showed an association between rsFC and future substance use, the results did not hold after controlling for SES and stress effects. Thus, it is still unknown if there is a significant association between rsFC and future substance use, and whether rsFC carries some of the effects of social and environmental factors on substance use behaviors. This suggests that while corticostriatal connectivity may contribute to cognitive and emotional regulation, it may predict substance use initiation because of the effects of SES and stress. Future studies should explore additional mediating factors, such as peer influence, environmental exposure, and genetic predispositions, to better understand the complex pathways leading to substance use in youth.

### 4.1. Strengths

This study has several strengths, including the use of data from the Adolescent Brain Cognitive Development (ABCD) study, which enhances the generalizability of the findings. The longitudinal design provides an opportunity to explore potential predictive relationships between baseline connectivity and future behavioral outcomes. Additionally, the use of rs-fMRI provides an objective assessment of neural connectivity, which may reduce reliance on self-reported behavioral data and offer insights into the neurobiological mechanisms underlying health disparities.

### 4.2. Limitations

Despite these strengths, several limitations should be considered. The observational nature of the study precludes causal interpretations of the observed associations. Additionally, while efforts were made to control for potential confounders, unmeasured factors such as genetic predispositions and environmental influences may have influenced the results. The reliance on self-reported measures for substance use and trauma exposure introduces the potential for recall bias. Moreover, the effect sizes observed in the study were small, suggesting that additional factors beyond corticostriatal connectivity contribute to the examined outcomes.

### 4.3. Future Research Directions

Future research should explore whether interventions aimed at enhancing corticostriatal connectivity could support cognitive and emotional development in youth exposed to socioeconomic and environmental disadvantages. Longitudinal studies with repeated neuroimaging assessments may provide further insight into the developmental trajectories of corticostriatal connectivity. Additionally, incorporating protective factors such as social support and educational enrichment programs may help mitigate the adverse effects of socioeconomic disparities on neural and behavioral outcomes.

## 5. Conclusion

In conclusion, this study suggests that corticostriatal connectivity, particularly between the left caudate and the cingulo-opercular network, may be associated with cognitive and behavioral outcomes in youth. Lower connectivity was linked to socioeconomic and environmental disadvantages, as well as cognitive performance and impulsivity. However, the study found no significant association with past depression or future substance use, suggesting that other factors may play a more critical role in these outcomes. These findings underscore the importance of considering both social and neurobiological factors when addressing health disparities in youth. Further research is needed to establish causal mechanisms and inform targeted interventions aimed at promoting resilience and reducing disparities in behavioral health outcomes.

## Figures and Tables

**Figure 1.**
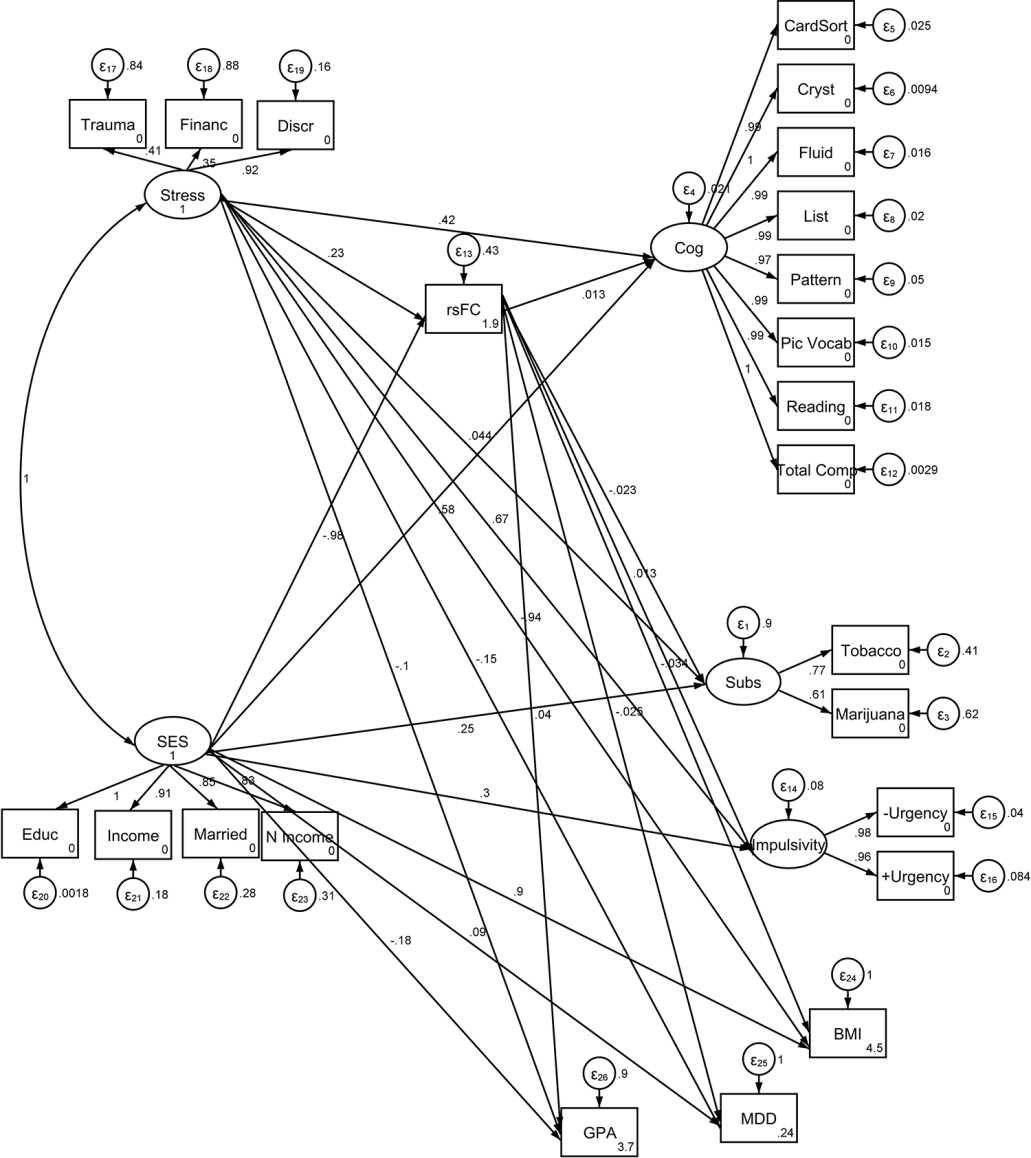
Bivariate correlations between study variables Note: BMI= Body Mass Index; GPA= Grade Point Average; N Income: Neighborhood Income; rsFC = Left Caudate Resting-State Functional Connectivity with the Cingulo-Opercular Network; SES= Socioeconomic Status.

**Table 1.** Bivariate correlations between study variables

	1	2	3	4	5	6	7	8	9	10	11	12	13	14	15	16	17	18	19	20	21	22	23	24	25	26	27
1 rsFC	1.00																										
2 Trauma (n)	−0.03	1.00																									
3 Financial Strain (Mean)	−0.03	0.16	1.00																								
4 Parental Education	0.02	−0.06	−0.28	1.00																							
5 Income to Need	0.02	−0.08	−0.30	0.48	1.00																						
6 N Income / 50000	0.04	−0.07	−0.22	0.39	0.31	1.00																					
7 Discrimination	−0.03	0.05	0.15	−0.17	−0.15	−0.14	1.00																				
8 Body Mass Index (BMI	−0.04	0.04	0.16	−0.23	−0.13	−0.18	0.08	1.00																			
9 Married Household	0.03	−0.11	−0.24	0.28	0.10	0.25	−0.11	−0.16	1.00																		
10 Negative Urgency.	−0.02	0.03	0.06	−0.05	−0.06	−0.03	0.12	0.01	−0.04	1.00																	
11 Positive Urgency	−0.03	0.05	0.11	−0.13	−0.11	−0.09	0.15	0.04	−0.09	0.50	1.00																
12 MDD (Past)	−0.02	0.02	0.05	−0.04	−0.02	−0.04	0.10	0.04	−0.03	0.09	0.09	1.00															
13 Drive	−0.01	0.02	0.09	−0.15	−0.08	−0.12	0.14	0.09	−0.10	0.21	0.24	0.07	1.00														
14 GPA (1–5)	0.03	0.01	−0.01	−0.05	−0.06	−0.04	−0.03	0.03	−0.01	−0.02	−0.03	−0.02	−0.02	1.00													
15 Cognition (Card Sort)	0.03	−0.03	−0.11	0.19	0.12	0.15	−0.10	−0.10	0.12	−0.05	−0.10	−0.03	−0.10	0.05	1.00												
16 Cognition (Crystalized)	0.02	−0.05	−0.20	0.40	0.32	0.27	−0.20	−0.14	0.23	−0.10	−0.17	−0.05	−0.18	0.01	0.26	1.00											
17 Cognition (Fluid Composition)	0.03	−0.04	−0.14	0.26	0.17	0.21	−0.14	−0.12	0.17	−0.08	−0.14	−0.04	−0.15	0.05	0.71	0.40	1.00										
18 Cognition (List)	0.02	−0.04	−0.13	0.29	0.19	0.20	−0.14	−0.11	0.17	−0.06	−0.12	−0.04	−0.14	0.02	0.26	0.43	0.59	1.00									
19 Cognition (Pattern)	0.03	−0.02	−0.07	0.11	0.07	0.10	−0.05	−0.04	0.08	−0.04	−0.09	−0.02	−0.08	0.04	0.41	0.18	0.72	0.19	1.00								
20 Cognition (Picture Vocabulary)	0.02	−0.05	−0.19	0.39	0.31	0.28	−0.19	−0.13	0.23	−0.10	−0.16	−0.04	−0.18	−0.03	0.23	0.85	0.35	0.38	0.16	1.00							
21 Cognition (Reading)	0.02	−0.04	−0.15	0.30	0.24	0.18	−0.15	−0.11	0.17	−0.07	−0.13	−0.03	−0.13	0.04	0.22	0.88	0.35	0.36	0.16	0.50	1.00						
22 Cognition (Total Composition)	0.03	−0.05	−0.20	0.40	0.30	0.29	−0.20	−0.16	0.24	−0.10	−0.18	−0.05	−0.19	0.03	0.57	0.85	0.83	0.61	0.53	0.72	0.74	1.00					
23 Tobacco Use (Subsequent)	−0.03	0.04	0.04	−0.04	−0.02	0.00	0.05	0.05	−0.04	0.04	0.04	0.03	0.03	0.01	−0.01	−0.02	−0.02	−0.03	0.00	−0.02	−0.01	−0.02	1.00				
24 Marijuana Use (Subsequent)	−0.01	0.03	0.06	−0.06	−0.03	−0.03	0.05	0.07	−0.06	0.03	0.04	0.03	0.02	−0.01	−0.01	−0.01	−0.01	−0.01	0.00	−0.01	−0.01	−0.01	0.45	1.00			
25 Tobacco and Marijuana (Subsequent)	−0.01	0.03	0.05	−0.03	−0.03	−0.01	0.03	0.06	−0.05	0.03	0.04	0.02	0.02	0.00	0.00	0.00	−0.01	−0.01	0.01	0.00	−0.01	−0.01	0.60	0.76	1.00		
26 Tobacco Only (Subsequent)	−0.02	0.03	0.02	−0.03	0.00	0.01	0.04	0.02	−0.02	0.02	0.02	0.02	0.03	0.02	−0.01	−0.02	−0.02	−0.03	−0.01	−0.02	−0.01	−0.03	0.78	−0.03	−0.03	1.00	
27 Marijuana Only (Subsequent)	−0.01	0.01	0.04	−0.05	−0.02	−0.02	0.04	0.04	−0.04	0.01	0.02	0.01	0.01	−0.01	−0.02	−0.01	−0.01	−0.01	0.00	−0.02	0.00	−0.01	−0.03	0.64	−0.02	−0.02	1.00

**Table 2.** Summary of SEM results

Structural	B	SE	95% CI		p
rsFC					
Stress	0.229	0.019	0.193	0.265	< 0.001
SES	−0.983	0.029	−1.039	−0.927	< 0.001
Intercept	1.911	0.042	1.828	1.993	< 0.001
					
BMI					
rsFC	−0.034	0.016	−0.065	−0.003	0.029
Stress	−0.943	0.128	−1.194	−0.691	< 0.001
SES	0.901	0.051	0.801	1.001	< 0.001
Intercept	4.518	0.144	4.235	4.800	< 0.001
					
MDD (Past)					
rsFC	−0.025	0.017	−0.058	0.009	0.146
Stress	−0.155	0.338	−0.816	0.507	0.647
SES	0.090	0.405	−0.704	0.884	0.824
Intercept	0.243	0.175	−0.100	0.587	0.165
					
GPA					
rsFC	0.040	0.024	−0.007	0.086	0.098
Stress	−0.101	0.312	−0.712	0.510	0.746
SES	−0.184	0.404	−0.976	0.607	0.648
Intercept	3.742	0.070	3.605	3.879	0.000
					
Substance Use					
rsFC	−0.023	0.017	−0.057	0.010	0.174
Stress	0.044	0.369	−0.679	0.767	0.905
SES	0.252	0.373	−0.479	0.983	0.500
					
Cognitive Function					
rsFC	0.013	0.003	0.007	0.019	< 0.001
Stress	0.415	0.003	0.409	0.421	< 0.001
SES	0.583	0.002	0.578	0.587	< 0.001
					
Impulsivity					
rsFC	0.013	0.006	0.001	0.025	0.034
Stress	0.671	0.008	0.655	0.687	< 0.001
SES	0.297	0.005	0.287	0.307	< 0.001

Note: BMI= Body Mass Index; GPA= Grade Point Average; rsFC = Left Caudate Resting-State Functional Connectivity with the Cingulo-Opercular Network; SES= Socioeconomic Status.

**Table 3.** Summary of measurements in the SEM model

	B	SE	95% CI		p
Measurement					
Substance Use					
Tobacco	0.767	.0191	0.730	0.805	< 0.001
Marijuana	0.614	.0161	0.583	0.646	< 0.001
Cognition					
Card Sort	0.987	.0003	0.987	0.988	< 0.001
Crystal	0.995	.0001	0.995	0.996	< 0.001
Fluid Comp	0.992	.0002	0.992	0.992	< 0.001
Total Comp	0.999	.0000	0.998	0.999	< 0.001
Pattern	0.975	.0005	0.974	0.976	< 0.001
Pict Vocab	0.993	.0002	0.992	0.993	< 0.001
Reading	0.991	.0002	0.990	0.991	< 0.001
List	0.990	.0002	0.989	0.990	< 0.001
Impulsivity					
Negative Urgency	0.980	.0008	0.978	0.981	< 0.001
Positive Urgency	0.957	.0013	0.954	0.960	< 0.001
Stress					
Trauma	0.406	.0074	0.391	0.421	< 0.001
Financial Difficulty (n)	0.347	.0077	0.332	0.362	< 0.001
Discrimination	0.918	.0020	0.914	0.922	< 0.001
SES					
Education (Jaeger)	0.999	.0002	0.999	1.000	< 0.001
Income to Needs	0.907	.0017	0.904	0.910	< 0.001
Married partnered	0.849	.0027	0.844	0.854	< 0.001
N Income / 5000	0.832	.0029	0.827	0.838	< 0.001

Note: BMI= SES= Socioeconomic Status; N Income: Neighborhood Income
